# A review of studies evaluating the effectiveness of risk minimisation measures in Europe using the European Union electronic Register of Post‐Authorization Studies

**DOI:** 10.1002/pds.4434

**Published:** 2018-04-16

**Authors:** Pareen Vora, Esther Artime, Montse Soriano‐Gabarró, Nawab Qizilbash, Vineet Singh, Alex Asiimwe

**Affiliations:** ^1^ Epidemiology Department Bayer AG Berlin Germany; ^2^ OXON Epidemiology Madrid Spain; ^3^ London School of Hygiene and Tropical Medicine London UK; ^4^ Bayer AG Berlin Germany

**Keywords:** Europe, medical records, pharmacoepidemiology, review, risk management, surveys and questionnaires

## Abstract

**Purpose:**

An important element of risk management is the planning and implementation of risk minimisation measures (RMMs) and the evaluation of their effectiveness by process or outcome indicators. The aim of this review is to summarize the characteristics of risk minimisation (RM) effectiveness studies in Europe and provide an overview of RMMs and their effectiveness.

**Methods:**

This was a qualitative review of RM effectiveness studies in the European Union electronic Register of Post‐Authorization Studies (EU PAS Register); data extracted included study design, population, sample size, data sources, drug information, RMMs, study period, indicators, and their reported effectiveness.

**Results:**

Of the 872 records in the EU PAS Register, 19 studies evaluating the effectiveness of RMMs were included. Eleven were cross‐sectional surveys and 8 used secondary data sources. Eighty‐nine percent (17/19) evaluated additional RMMs (used when routine RMMs are considered insufficient), and 36% (7/19) evaluated changes in routine RMMs (applicable to all medicinal products). A total of 42 effectiveness indicators were identified: 18 process and 24 outcomes. Half of the indicators (21/42) were successful; 2% (1/42) indicators were partially successful; 17% (7/42) indicators were inconclusive. Effectiveness of the remaining 31% (13/42) indicators could not be determined owing to limited information. The United Kingdom was the most frequent country for the conduct of RM effectiveness studies.

**Conclusions:**

Most of the included studies evaluated additional RMMs. Half of the effectiveness indicators (process and/or outcome) were reported as successful. This review provides evidence to support the development of future guidance on the effectiveness of RM in Europe.

**KEY POINTS**

• The EU PAS Register is a valuable resource to identify post authorization studies evaluating the effectiveness of risk minimisation measures in Europe, for which study protocols and reports are available.
• This review summarizes the different routine and additional risk minimisation measures assessed in risk minimisation effectiveness studies.
• This review provides an overview of the different process and outcome indicators used to assess the effectiveness of risk minimisation measures.
• The majority of included studies did not pre‐specify a threshold for success.
• This review provides evidence to support further development of the guidance.

## 1 INTRODUCTION

A new Directive and Regulation (Directive 2010/84/EU and Regulation (EU) No 1235/2010) was adopted by the European Parliament in December 2010 bringing significant changes in the safety monitoring of medicines across the European Union (EU). The new Pharmacovigilance (PhV) legislation, which came into effect in July 2012, introduced significant changes around PhV processes in Europe including the release of 16 guideline modules outlining good PhV practices (GVP).^1^, ^2^ Module XVI was first adopted in 2014 to provide guidance for the selection and evaluation of the effectiveness of risk minimisation measures (RMMs). In the same year, the Council for International Organizations of Medical Sciences Working Group IX published Practical Approaches to Risk Minimisation, which provides a framework for the evaluation of effectiveness of RMMs.^3^

According to GVP module XVI, “RMMs are interventions intended to prevent or reduce the occurrence of adverse drug reactions associated with the exposure to a medicine or to reduce their severity or impact on the patient, should adverse reactions occur”.^4^ Marketing authorization holders are required to monitor the outcome of RMMs, which are included in the risk management plan (RMP) or as a condition of market authorization. The RMMs may be classified as routine (rRMMs) or additional risk minimisation measures (aRMMs). The rRMMs are applicable to all medicinal products, and the majority of safety concerns are adequately addressed by rRMMs. When rRMMs may not be sufficient, aRMMs may be required to manage and mitigate the risk(s) that supplement rRMMs.^4^

Effectiveness of RMMs is mainly evaluated for aRMMs but also sometimes for rRMMs. The effectiveness of RMMs can be evaluated by process and/or outcome indicators. Process indicators measure the extent to which a programme was implemented, whether the execution was as planned, and the impact on knowledge and behaviour of the target population. Outcome indicators provide an overall measure of the level of risk control achieved by RMM, for example, measuring rates of an adverse drug reaction or other safety‐related outcome.^4^ Evaluation of effectiveness of RMMs is important to manage the benefit‐risk balance of a medicinal product. Effectiveness of RMMs can be evaluated by using cross‐sectional survey studies and studies using secondary data sources.

The EU electronic Register of Post‐Authorization Studies (EU PAS Register) available through the European Network of Centres for Pharmacoepidemiology and Pharmacovigilance (ENCePP), launched in 2010, is a publicly available register of non‐interventional post‐authorization studies. The ENCePP activity report presented a rise in the registration of studies in the EU PAS Register from 20 studies in January 2012 and 440 in December 2014 to 968 in December 2016.^5^, ^6^, ^7^ The EU PAS Register includes study documents such as study protocol and report of the registered studies (based on status—planned, ongoing, or completed), which provides a unique opportunity to examine study details. According to the GVP VIII, marketing authorization holders are legally required to register non‐interventional post‐authorisation safety studies (PASS) imposed as an obligation (ie categories 1 and 2). It is also recommended to register all category 3 (required in the RMP) non‐interventional PASS and any other PASS to support transparency and facilitate exchange of information between different stakeholders.^8^ Therefore, the EU PAS Register is a valuable resource for PASS, including those evaluating the effectiveness of RMMs^9^ and those mandated by the European Medicines Agency. A review conducted by Gridchyna et al^10^ using MEDLINE and Embase included published studies evaluating the effectiveness of RMMs worldwide up to 2013, which was before GVP XVI was adopted. However, a comprehensive review of studies assessing the effectiveness of RMMs in European countries using EU PAS register is lacking including the studies initiated after GVP XVI. Therefore, the aim of this review is to describe and summarize PASS evaluating the effectiveness of RMMs in Europe and provide an overview of the RMMs and their effectiveness.

## 2 METHODS

This study was a qualitative review of RMM effectiveness studies using the EU PAS Register. All studies registered in the EU PAS Register from 2010 to 30 August 2016 were screened. Study titles were screened to identify those assessing the effectiveness of RMMs (hereinafter “RM effectiveness studies”) using keywords such as “minimisation,” “survey,” “effectiveness,” “drug utilization,” “behaviour,” “knowledge,” “materials,” and “physician.” Studies were reviewed using information provided in the register. If there was any ambiguity of eligibility, the study protocol and/or report was reviewed. Studies were included if they evaluated the effectiveness of RMM(s), were conducted in at least one European country, and a report or executive summary was available in the EU PAS Register. The screening process and data extraction were conducted by 2 independent reviewers (P. V. and E. A.), and discrepancies were discussed and resolved. For the purpose of data extraction, the final version of study reports was used. When the study report was absent, the executive summary was used. All references were managed by using EndNote X7 (Thomson Reuters, USA). Table 1 summarizes the variables abstracted from the included studies and their corresponding operational definitions.

**Table 1:** Variables abstracted from studies evaluating the effectiveness of RMMs in Europe

Variable	Operational Definition
Study type	Surveys using primary data collection or studies using secondary data
Target population (survey studies)	Patients, specialists, GPs, or other HCPs
Source for recruitment	Panel(s) or list(s) of prescribers used for recruitment
Countries and no. of subjects/patients	Participating countries in each study plus number of subjects/patients from each and in total
Data source (secondary data studies)	Secondary data sources used for evaluating RMM(s) including chart reviews.
Risk and drug	Risk related to the drug for which the RMM(s) was implemented
Indication	Indication of the drug for which the RMM is intended
RMMs	As per the GVP module XVI^4^: rRMMs: SmPCs, package leaflet, labelling, pack size and design, legal status of the product aRMMs: Educational programmes/tools for patients or HCPs, controlled access programmes, controlled distribution systems, PPPs, and DHPC.
Indicators	Process indicators: implementation and receipt of the RMM (eg educational materials reaching the target group and change in knowledge), understanding and awareness of HCPs or patients (eg knowledge gained by physicians about the importance of metabolic monitoring), and behavioural change (eg actual proportions of testing conducted by physicians). Outcome indicators: rates of an adverse drug reaction or other safety‐related outcome; (eg reduction in the incidence of risk under consideration after the implementation of RMM). *Note: One or more indicators could be assessed to evaluate the effectiveness of a single RMM*.^4^
Implementation date	Date of implementation of the RMM
Study period	The period of data collection (for surveys); the period for which the data were analyzed (for studies using secondary data sources).
Reported effectiveness (for each individual indicator)	Successful: the indicator assessing RMM achieved a pre‐specified threshold, the study concluded that the RMM was successful, no further RMM was required, RMM was sufficient, or used similar terms. Inconclusive: the indicator assessing RMM did not achieve a pre‐specified threshold, the study concluded that the results provided insufficient evidence, further analysis was required, or similar terms. *Note: these categories were ascertained solely based on the study results and conclusions in the study report*.

Abbreviations: aRMM, additional risk minimisation measure; DHPC, Dear Healthcare Professional Communications; GP, general practitioner; GVP, good pharmacovigilance practices; HCP, health care professional; PPPs, pregnancy prevention programmes; rRMM, routine risk minimisation measure; RMM, risk minimisation measure; SmPC summary of product characteristics.

## 3 RESULTS

**Figure 1:**
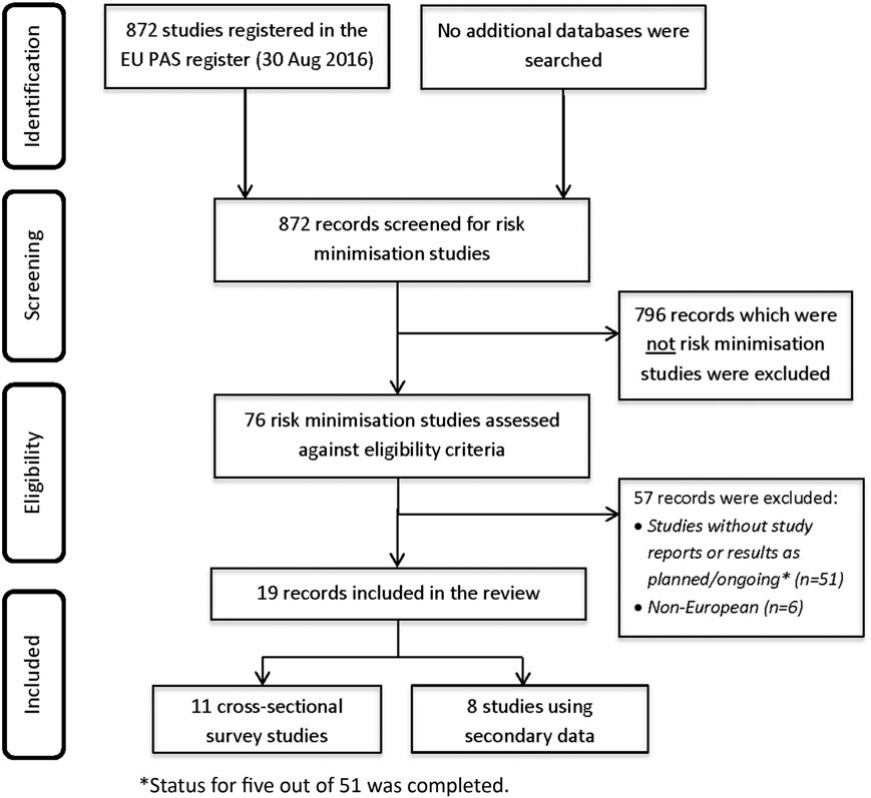
PRISMA flow diagram

The screening process and the final number of studies included in the review are shown in Figure 1 **.** Of the 872 studies registered in the EU PAS register from 2010 to 30 August 2016, 76 were RM studies with status planned, ongoing, or completed. In total, 19 RM effectiveness studies met the selection criteria and were included in this review.^11^, ^12^, ^13^, ^14^, ^15^, ^16^, ^17^, ^18^, ^19^, ^20^, ^21^, ^22^, ^23^, ^24^, ^25^, ^26^, ^27^, ^28^, ^29^

### 3.1 Cross‐sectional surveys

Eleven of the 19 included studies were cross‐sectional surveys of medical specialists, general practitioners, or other health care professional (HCPs,^12^, ^13^, ^14^, ^15^, ^16^, ^21^, ^23^, ^24^, ^25^ and 2 also included patients and caregivers.^22^, ^29^ Ten surveys had a cross‐sectional design, and one had a cross‐sectional quasi‐experimental design.^16^ All surveys were voluntary, and the participants were contacted/recruited by email, post, or via telephone to participate. Of the 11 surveys, 3 used a network or an established panel of HCPs,^12^, ^14^, ^15^ 4 targeted prescribers/potential prescribers who were sent RM materials,^13^, ^16^, ^21^, ^24^ 1 selected patients and caregivers who received RM materials,^29^ and the remaining 3 randomly selected prescribers or potential prescribers^22^, ^23^, ^25^ of which 1 also randomly selected treated patients.^22^ Eight were conducted as online surveys,^12^, ^13^, ^14^, ^15^, ^16^, ^21^, ^24^, ^25^ 1 involved face to face interviews of prescribing physicians and treated patients,^22^ 1 involved direct observation of patients and caregivers,^29^ and 1 was conducted via mail/telephone.^23^ The number of included countries ranged from 5 to 10 per survey except 1 survey that was conducted in 1 country.^23^ The countries most frequently included (≥5 studies) were as follows: the United Kingdom (10/11), Spain (9/11), Denmark (8/11), Germany (7/11), France (5/11), Netherlands (5/11), and Sweden (5/11). Figure S1 shows the most frequently included EU countries in the surveys. Two of the 11 surveys also included non‐EU countries, which were Switzerland^13^, ^24^ and Hong Kong.^24^ The total number of participants in the 9 cross‐sectional surveys was based on quoted sample size estimations and ranged from 250 to 802, except 1 survey that involved 1 country that recruited 32 prescribers^23^ and 1 multi‐country study that recruited 40 patients or caregivers.^29^ The range of participants per country was 2 to 212.

Two of 11 surveys evaluated routine RMMs (SmPC)^12^, ^25^ and 9 evaluated aRMMs, which included Direct Healthcare Professional Communications (DHPC), physician's guides, checklists, educational materials, and patient alert cards. These RMMs were evaluated by using process indicators such as receipt of materials, understanding, knowledge, awareness, utilization, and behaviour. In total, there were 19 indicators assessing RMMs of which 13 were successful, ie effective. The remaining 6 indicators were inconclusive, of which 1 indicator was understanding and knowledge of the physician's guide,^13^ 1 was receipt of educational materials by HCPs,^16^ and the other 4 indicators were awareness, use, knowledge, and behaviour of using the checklist, Q&A brochure, and patient alert card.^21^ All countries planned for the survey eventually participated in the study except for 1 study where the survey was not initiated in one country (Sweden).^13^ Two studies planned to include both HCPs and patients; however, one study could not recruit patients owing to confidentiality regulations.^13^ Table 2 summarizes the characteristics of the cross‐sectional surveys evaluating the effectiveness of RMMs and their reported effectiveness.

**Table 2:** Characteristics of cross‐sectional survey studies

No.	Target Population	Source	Countries	No. of Subjects	Drug	Indication	Risk	RMM Under Evaluation	Indicator Assessing the Effectiveness of RMM	Indicator Type	RMM Implementation Time	Study Period	Reported Effectiveness of RMM
1^15^, ^a^	GPs and specialists (paediatricians, child/adolescent psychiatrists, and other non‐paediatrician psychiatrists)	Invitation to prescribers in the distribution list of DHPC and panel of HCPs	DK	100	Atomoxetine	ADHD	Cardiovascular and cerebrovascular risk (paediatric patients)	DHPC (SmPC changes, physician guide, associated checklists, and measurement recording chart)	(Wave 2) Reassess^g^ knowledge, awareness, and adherence to changes	Process	2011	2013	Successful
			SE	50									
			NL	100									
			ES	100									
			UK	200									
			Total	550									
2^14^, ^a^	Specialists (psychiatrists)	Panel of HCPs	DK	30	Atomoxetine	ADHD	Cardiovascular and cerebrovascular risk (adult patients)	SmPC changes, physician guide, associated checklists, and measurement recording chart)	Knowledge, awareness, and adherence to changes	Process	2011	2014	Successful
			SE	40									
			NL	40									
			ES	70									
			UK	70									
			Total	250									
3^16^, ^a^	Specialists (psychiatrists and neurologists) GPs, and other HCPs	Invitation to physicians who were potential prescribers in the distribution list of the materials	UK	100	Quetiapine fumarate	Anti‐psychotic	Weight gain, hyperglycaemia, and worsening of lipid profile	Educational materials	Receipt educational materials	Process	2012	2013	Inconclusive
			DE	100									
			IT	100									
			RO	100									
			ES	100					Behaviour regarding monitoring of specific metabolic parameters	Outcome^h^			Successful
			SE	100									
			HU	100									
			AT	100									
			Total	800									
4^29^, ^b^	Patients and caregivers	Patients or caregivers who received HAT pack	AT	NS	Romiplostim	Immune thrombo‐cytopenic purpura	Medication errors from self‐administration	Home administration training pack	Administered romiplostim correctly	Outcome	Dec 2012	7 Jul 2014 to 20 Jan 2016	Successful
			BE	NS									
			FR	NS									
			DE	NS									
			EL	NS									
			NL	NS									
			ES	NS									
			UK	NS									
			Total	40									
5^23^, ^c^	GPs	Random sample of physicians working in general practice	DK	32	Cyproterone acetate and ethinilstradiol	Moderate to severe androgen‐sensitive acne without seborrhoea and/or hirsutism in women of reproductive age	New safety precautions; adjustment of indication; contraindications; thromboembolic complications	DHPC and educational material	Knowledge	Process	Jun 2013	2015	Successful
			**Total**	**32**									
6^13^, ^a^	Specialists (neurologists) and patients^e^	Invitation to prescribers in the distribution list of the physician guide	DE	96	Retigabine	Partial‐onset seizures	Prolongation of QT interval, voiding dysfunction/urinary retention, and hallucinations/confusion/psychotic disorders	Physician guide	Understanding and knowledge	Process	Not in the report	2012–2013	Inconclusive
			DK	15									
			UK	53									
			CH	23									
			SE^e^	‐									
			ES	60									
			SK	28									
			NO	19									
			Total	294									
7^24^, ^a^	Specialists (general neurology, neurosurgery, neuro‐psychiatry, and epileptologists)	Invitation to prescribing and non‐prescribing physicians in the DHPC distribution list	BE	51	Retigabine	Epilepsy	Eye disorders, ie pigment changes, skin, and subcutaneous disorders	DHPC	Awareness and knowledge	Process	Jun 2013	2014–2015	Successful
			HK	2									
			NO	17									
			SK	66									
			ES	186									
			CH	29									
			UK	63									
			Total	414									
8^21^, ^a^	Specialists (critical care, haematology, infectious diseases, intensive care, microbiology, and oncology, transplant)	Invitation to prescribers who received the RMMs	AT	2	Voriconazole	Fungal infections	Phototoxicity, squamous cell carcinoma, and hepatic toxicity	HCP checklist, HCP question and answer brochure, and patient alert card	Awareness (receipt)	Process	Apr 2014	2015–2016	Inconclusive^i^
			DK	5									
			FR	42									
			DE	16					Utilization	Process			Inconclusive
			HU	13									
			IE	7									
			IT	14					Knowledge	Process			Inconclusive i
			NL	21									
			ES	191									
			UK	21					Behaviour	Process			Inconclusive
			Total	332									
9^12^, ^a^	Specialists (HIV, infectious disease, and genito‐urinary), GPs, nurses, and pharmacists	Panels of HIV prescribers	AT/DE	101	Rilpivirine and Emtricitabine/Rilpivirine/Tenofovir disoproxil fumarate	HIV‐1 infection	Lack of therapeutic effect potentially leading to development of resistance when taking the drug without food/meal	SmPC^f^ (prescribing information)	Understanding	Process	Not applicable	2014	Successful
			BE/NL	49									
			FR	71									
			UK	73					Utilization of prescribing instructions	Process			Successful
			NO	6									
			DK	7									
			SE	16									
			Total	323									
10^25^, ^a^	Specialists (oncologists)	Random sample of oncologists selected from the master list for each country and stratified by region	DK	NS	Denosumab	Anti‐resorptive therapy in patients with advanced cancer	Osteonecrosis of the jaw	SmPC^f^	Knowledge	Process	Not‐ applicable	(2 rounds) 2013–2014 and 2013–2015	Successful
			FI	NS									
			FR	NS									
			DE	NS									
			IT	NS									
			NO	NS									
			ES	NS									
			SE	NS									
			UK	NS									
			Total	420									
11^22^ d	Specialists (cardiologists) and GPs	Prescribing HCPs randomly selected	BG	58	Dabigatran	SPAF	Bleeding	Prescriber guide and patient alert card (within the educational pack to physicians)	Receipt and distribution to patients	Process	Not in the report	2015	Successful
			CZ	64									
			DK	1									
			FR	50					Knowledge and recommendations to their AF patients	Process			Successful
			DE	69									
			SK	46									
			ES	62									
			UK	61									
			Total	411									
	Patients	Treated patients randomly selected	BG	103				Patient alert card (within the pack)	Receipt	Process	Not in the report	2015	Successful
			CZ	108									
			DK	43									
			FR	92					Understanding	Process			Successful
			DE	212									
			SK	59									
			ES	118									
			UK	67									
			Total	802									

a Online survey.

b Data collected using direct observation.

c Survey by mail or telephone.

d Face to face survey.

e Initially planned but not included in the study after challenges encountered.

f Routine risk minimisation measure.

g First risk minimisation effectiveness assessment survey was conducted in 2012 (Wave 1).

h As reported in the study.

i Successful for most but not all risks.

Abbreviations: ARV, antiretroviral; ADHD, attention deficit hyperactivity disorder; AF, Atrial Fibrillation; DHPC, Direct Healthcare Professional Communication; GPs, general practitioners; HIV‐1, human immunodeficiency virus type 1; NS, not specified; RMMs, risk minimisation measures; SmPC, summary of product characteristics; SPAF, stroke prevention for atrial fibrillation; HAT, home administration training.

Countries: AT, Austria; BE, Belgium; BG, Bulgaria; HR, Croatia; CZ, Czech Republic; DK, Denmark; EE, Estonia; FI, Finland; FR, France; EL, Greece; DE, Germany; HU, Hungary; HK, Hong Kong; IE, Ireland; IT, Italy; LV, Latvia; LT, Lithuania; LU, Luxembourg; NL, Netherlands; PL, Poland; PT, Portugal; RO, Romania; SI, Slovenia; SK, Slovakia; ES, Spain; SE, Sweden; CH, Switzerland; UK, United Kingdom.

### 3.2 Studies using secondary data sources

Eight of the 19 included studies used secondary data sources for RM effectiveness studies.^11^, ^17^, ^18^, ^19^, ^20^, ^26^, ^27^, ^28^, ^29^ Two of the 8 studies involved chart review using electronic medical records.^11^, ^17^ The remaining 6 studies used multiple health care databases, which included Aarhus University Research Database and population health registers from Denmark, Clinical Practice Research Datalink (CPRD) from the United Kingdom, Integrated Primary Care Information , Dutch PHARMO general practitioner database from the Netherlands, and the Emilia Romagna regional database from Italy. The countries most frequently included (≥2 studies) were United Kingdom (7/8 studies), Denmark (2/8 studies), Germany (2/8 studies), and Netherlands (2/8 studies). Among the 7 studies involving United Kingdom, 5 studies used CPRD.^18^, ^19^, ^20^, ^26^, ^27^ Table 3 summarizes the characteristics of RM effectiveness studies using secondary data sources, and Figure S2 shows the countries where these studies were conducted. Seven were retrospective cohort studies,^11^, ^18^, ^19^, ^20^, ^26^, ^27^, ^28^ and 1 was cross sectional.^17^ All 7 retrospective studies were drug utilization studies of which 3 had a pre‐post design.^11^, ^19^, ^26^ The total sample size in the 7 retrospective cohort studies ranged from 687 to 34 975.^11^, ^18^, ^19^, ^20^, ^26^, ^27^, ^28^ One cross‐sectional study used electronic medical records from the IMS® Disease Analyzer and included 294 subjects.^17^

**Table 3:** Characteristics of studies using secondary data sources

No.	Design	Countries	No. of Patients	Database/Data Source	Drug	Indication	Risk	RMMs Under Evaluation	Indicator Assessing the Effectiveness of RMMs	Indicator Type	RMM Implementation Time	Study Period	Reported Effectiveness
12^20^ ^a^	Retrospective cohort study (DUS)	DK	2321	Population health registries of 2 northern regions	Rosiglitazone containing products	Type 2 diabetes mellitus	Risk of cardiovascular outcomes	Labelling changes^b^ and suspension from EU markets	Utilization dynamics	Outcome	23 Sep 2010	2000–2010	aLimited information^a^
		UK	25 428	GPRD (CPRD)					% of users with contraindications	Outcome			Limited information^a^
									Glycaemic control and other parameters	Outcome			Limited information^a^
		Total	27 749	‐									
13^18^	Retrospective cohort study (DUS)	UK^c^	323	GPRD (CPRD)	Isotretinoin	Severe nodular acne vulgaris that is unresponsive to other, first‐line therapies	Teratogenic effects	Pregnancy prevention programme	Prescription during pregnancy	Outcome	Not in the report	Jan 2004 to Dec 2010	Inconclusive
		UK Wales^d^	‐	SAIL									
		IT	5882	Emilia Romagna regional database									
		IT^d^	‐	Tuscany regional database									
		DK^d^	‐	Statistik Denmark									
		Total	6205	‐									
14^19^	Retrospective cohort study (DUS; pre/post‐design)	DK	878 new users	AURD	Pioglitazone	Type 2 diabetes mellitus (secondline therapy)	Bladder cancer	DHPC—to restrict use in patients without known risk factors of bladder cancer	Drug utilization patterns before and after DHPC	Outcome	Jul‐Aug 2011	1 Jan 2005 to 2 Feb 2012	Limited information
		NL	789 new users	IPCI					Events, ADRs, and diabetes control in discontinued patients after DHPC	Outcome		1 Jan 2007 to 30 Jun 2012	Limited information
		UK	33 308 new users	CPRD (former GPRD)					Contraindications, events, ADRs, and diabetes control in prevalent/new users after DHPC	Outcome		1 Jan 2000 to 31 Mar2012	Limited information
		Total	34 975	‐									
15^26^, ^a^	Retrospective cohort study (DUS; pre/post‐design)	UK	32 947	CPRD (former GPRD)	Pioglitazone	Type 2 diabetes mellitus (second‐line therapy)	Bladder cancer, heart failure, and need of monitoring of therapy benefits	EU SmPC^b^ and unspecified RMMs	Bladder cancer	Outcome	Jul 2011	Not in the report	aLimited information^a^
									Regular monitoring of therapy benefits	Outcome			Limited information^a^
		Total	32 947	‐					Prevalent heart failure	Outcome			Limited information^a^
16^28^, ^a^	Retrospective cohort study (DUS)	NL	2238	Dutch PHARMO GP database	Pioglitazone	Type 2 diabetes mellitus (second‐line therapy)	Bladder cancer and heart failure	SmPC changes^b^ and unspecified RMMs	Contraindications	Outcome	Jul 2011	2003–2011/2012	Successful
									Utilization	Outcome			Successful
									Monitoring frequencies	Outcome			Successful
		Total	2238	‐									
17^27^, ^a^	Retrospective cohort study (DUS)	UK	1808 new‐users; 12 986 prevalent users	CPRD (former GPRD)	Pioglitazone	Type 2 diabetes mellitus (second‐line therapy)	Heart failure, bladder cancer, acroscopic hematuria, and first‐line use	Label change^b^ and unspecified RMMs	First‐line use	Outcome	Jul 2011	21 Jul 2011 to 21 Dec 2013	Successful
									Incidence of heart failure	Outcome			Limited information^a^
									Bladder cancer	Outcome			Successful
									Macroscopic hematuria	Outcome			Limited information^a^
									Monitoring of glucose	Outcome			Limited information^a^
		Total	14 794	‐					Creatinine	Outcome			Limited information^a^
18^11^	Retrospective cohort study (DUS; pre/post‐design)	AT	101	Electronic medical records or paper registries from hospitals in each country	Tigecycline	Complicated intra‐abdominal Infection, complicated skin or soft tissue infection, and excluding diabetic foot infection	Super‐infection, lack of efficacy, and off‐label use	Changes to the SmPC^b^, DHPC, and educational material for HCPs	Incidence of superinfection	Outcome	Feb 2011	Pre: Feb 2010–2011; Post: Feb 2012–2013	Successful
		DE	315						Incidence of lack of efficacy cases	Outcome			Successful
		EL	27										
		UK	244										
		IT^d^	‐						Incidence of off‐label indication use	Outcome			Successful
		ES^d^	‐										
		Total	687^e^	‐									
19^17^	Cross‐sectional study	DE	1451	IMS disease analyzer (retrospectively collected EMRs)	Quetiapine fumarate	Antipsychotic	Weight gain, hyperglycaemia, and worsening of lipid profile	Educational materials	Evaluation and metabolic monitoring of patients	Outcome	Early 2012	13 Feb to 31 Aug 2012	Inconclusive in DE
		UK	887										
												11 Jan to 31 July 2012	Successful in UK
		Total	2338	‐									

a Only executive summary available.

b Routine risk minimisation measure.

c Feasibility study.

d Initially planned but not included in the study after challenges encountered.

e Sample size differed according to indicators evaluated.

Abbreviations: ADR, adverse drug reaction; AURD, Aarhus University Research Database; CPRD, Clinical Practice Research Datalink; DHPC, Direct Healthcare Professional Communication; EMRs, Electronic Medical Records; DUS, drug utilization study; GPRD, General Practice Research Datalink; IPCI, Integrated Primary Care Information; SAIL, Secure Anonymised Information Databank; SmPC, Summary of Product Characteristics; EU, European Union.

Countries: AT, Austria; BE, Belgium; DK, Denmark; FR, France; EL, Greece; DE, Germany; IT, Italy; NL, Netherlands; ES, Spain; UK, United Kingdom.

All 8 studies evaluated aRMMs that included educational materials, a pregnancy prevention programme, DHPCs; 5 studies also evaluated changes in rRMMs (SmPC/label changes).^11^, ^20^, ^26^, ^27^, ^28^ These RMMs were evaluated by using outcome indicators. Of the total of 23 indicators, success was achieved with 8 indicators,^11^, ^27^, ^28^ 1 was inconclusive,^18^ 1 study with 1 indicator achieved success in 1 country but was inconclusive in the other included country (partially successful),^17^ and success could not be determined due to limited reported information with remaining 13 indicators.^19^, ^20^, ^26^, ^27^ In general, the indicators examined changes in incidence of the risk under evaluation (n = 7), monitoring parameters (n = 7), drug use patterns (n = 5), pregnancy prevention (n = 1), and prescribing in patients with contraindications (n = 3). Three studies with 9 indicators used pre‐post design to evaluate effectiveness.^11^, ^19^, ^26^ Two studies were unable to use the planned data sources due to various challenges encountered.^11^, ^18^ These include medical records from Spain that required informed consent from all patients (deceased and living), medical records from Italy where physicians declined to participate owing to informed consent form requirements or lack of staff, Secure Anonymised Information Databank (SAIL) from the United Kingdom where ethics approval could not be obtained, General Practice Research Database where the study had to be carried out as feasibility study because Independent Scientific Advisory Committee claimed the study was not feasible, Tuscany regional database in Italy where the data could not be obtained in time, and Statistik Denmark from Denmark where it was identified that the required information for the study was limited.

### 3.3 Threshold

Two of 11 cross‐sectional surveys established a pre‐defined threshold for success, which was set as at least 80% of correct responses.^12^, ^21^ In one of the 2 studies, that defined threshold a priori, the selection of threshold was considered subjective and not based on prior knowledge, or established scientific criteria, and was also acknowledged by European Medicines Agency in their interactions.^21^ In the remaining 9 studies, a majority of participants correctly achieving the desired result were considered as successful. No study using secondary data sources defined success; however, 3 of the 8 studies used a pre‐post implementation of RMM design, in which success was judged by reduction in risk post RMM implementation.^11^, ^19^, ^26^

## 4 DISCUSSION

This review provides a qualitative overview of 11 cross‐sectional survey studies and 8 studies using secondary data evaluating the effectiveness of RMMs in EU using the EU PAS Register. A substantial number (~90%) of the included studies evaluated aRMMs. Most aRMMs communicated label changes. Indicators included process and/or outcome to assess the effectiveness of aRMMs in accordance with GVP module XVI; half of them achieved success. Typically cross‐sectional survey studies used process indicators to assess the effectiveness of RMMs, and studies using secondary data used outcome indicators of various kinds. While secondary data source studies mainly assessed label changes with or without DHPCs and educational materials in few cases, survey studies mostly evaluated educational materials and DHPCs. Two survey studies assessed the existing label to determine whether further actions are needed.^12^, ^25^ This review demonstrates the utilization of secondary data sources to conduct RM effectiveness studies in countries with health care data sources that are suitable for particular outcome measure. The United Kingdom was the most frequently selected country, included in all but 2 studies. Other frequent countries included Denmark, Spain, Germany, and Netherlands. More than two thirds of studies were multi‐country, and sample sizes varied considerably across studies. The choice of countries participating in RM effectiveness studies seems to be limited to a few that are consistently included in most evaluations (eg United Kingdom). This is particularly the case in studies using secondary data sources, hindering the extrapolation of results to other health care systems. It was also found that some countries initially planned to participate but were eventually excluded owing to challenges, and therefore, conducting feasibility assessment is very important. In a systematic review conducted by Gridchyna et al^10^ using MEDLINE and Embase, 65 published RM effectiveness studies worldwide were identified up to 2013; 19 of these were from Europe. None of the studies overlapped with those in this review, which was based on final study reports available in the EU PAS Register. Therefore, the results from this review supplement those presented by Gridchyna et al.

Data quality and completeness in cross‐sectional surveys is mostly dependent on the development and appropriateness of the survey questionnaire (eg cognitive interviews and linguistic validation) and the data collection method. Two in every 3 surveys in the review used online questionnaires, providing real‐time data efficiently and allowing the implementation of controls and data checks to enhance data quality (eg questions provided in a sequence, skipping patterns, and restricting changes). Also, survey studies are prone to recall, self‐report, and non‐response biases if participants who participate differ from those who do not, resulting in a non‐representative sample. However, outcomes for which studies using secondary data might not be feasible, survey studies can provide valuable insights. Few survey studies had disproportionate participation across countries, which may be a reflection of the usage of the product or difficulties identifying prescribers in these countries that could affect the generalizability of the results.^12^, ^21^, ^24^ Some studies encountered operational challenges such as access, approval, feasibility, and resources, which hindered their participation.^11^, ^18^ Sample size or the number of participants in survey studies can affect the results of the study if targeted number of participants is not achieved. Two studies in the review did not reach the pre‐specified sample size^13^, ^21^; of which one only recruited half of the planned number of participants and was inconclusive.^21^ Recruitment might be challenging in surveys and may limit generalizability (eg 1 survey study planned in patients and HCPs ‐ the patient survey part was excluded owing to confidentiality regulations).^13^ For studies using secondary data, quality of the data sources as well as validation of the outcomes is important to consider. Threshold for success of a RMM should be defined on a case‐by‐case basis considering the outcome, and a rationale for selection of the threshold should ideally be reported. While there should be strict threshold criteria for adverse events, for example, no pregnancy during exposure to teratogenic drugs, in other cases, the event cannot be mitigated and the RMM will only result in a reduction of the severity or aim to promote better control through monitoring or promoting action. However, few studies in this review reported a threshold for success and results were difficult to interpret for the others, highlighting the need for further guidance.

One potential approach for RM effectiveness could include survey component to evaluate process indicator(s) and secondary use of data to evaluate outcome indicator(s). One such example is included in this review, where physician's self‐reported behaviour (process indicator) was assessed through a cross‐sectional survey and monitoring of patients by physicians (outcome indicator) was assessed by using secondary data.^16^, ^17^ As these were 2 separate studies, no correlation of process and outcome indicators was reported.

The strengths of this review include the use of the EU PAS Register to review RM effectiveness studies and the availability of the detailed study reports. This review provides insights into RM effectiveness studies conducted in the EU with a good representation of potential study designs, target populations (physicians, nurses, pharmacists, and patients), practice settings (general practice and secondary care), RMMs (DHPC, pregnancy prevention programme, educational materials, and label changes), countries, data sources, and indicators (process and outcomes). This review also had some limitations. Firstly, the selection of studies may have been affected by publication bias. It is possible that category 3 PASS studies, which are required in the RMP or other PASS conducted voluntarily, for which registration in the EU PAS Register is not mandatory, might have been missed if not registered. However, out of the total 19 studies, 12 were category‐3^12^, ^13^, ^14^, ^16^, ^17^, ^21^, ^23^, ^24^, ^26^, ^27^, ^28^, ^29^ of which 10 were requested by regulatory authorities,^12^, ^14^, ^16^, ^17^, ^21^, ^23^, ^26^, ^27^, ^28^, ^29^ 3 studies were conducted by regulatory authorities,^18^, ^19^, ^20^ 1 was category‐2,^11^ and the remaining 3 were conducted voluntarily.^15^, ^22^, ^25^ Publication of these RM effectiveness studies should be encouraged as it adds important public health value. Additionally, 57 RM studies were excluded in the selection process because they were planned or ongoing—5 of these had status “finalized” but results were unavailable. Secondly, there were many studies with one or more risks and RMMs, but it was not possible to report on them separately as effectiveness was reported per indicator for the RMM(s) as a whole.. Thirdly, the success or effectiveness of the RMMs is based on the reported results and conclusions; however, its regulatory impact and consequences are unknown. Fourthly, only a summary report or abstract was available for 5 of the 19 included studies from which limited information could be extracted.^20^, ^26^, ^27^, ^28^, ^29^

## 5 CONCLUSIONS

Most of the included studies evaluated aRMMs, and some also evaluated rRMMs. Cross‐sectional surveys were used to assess process indicators, while studies using secondary data sources were designed to assess outcome indicators. Half of the effectiveness indicators (process and/or outcome) were reported as successful; however, to draw conclusions that could be extrapolated to future RM effectiveness studies, a quantitative assessment of study results is necessary. The EU PAS Register proves to be a valuable resource for identifying studies evaluating the effectiveness of RMMs in Europe. It shows the impact of GVP legislation on transparency, and it is likely to stimulate further discussions in this field. This review provides valuable information to further define areas where guidance for the design, methods, interpretation, and use of data sources are required to conduct RM effectiveness studies with high quality standards.

## CONFLICT OF INTEREST

P. Vora, A. Asiimwe, and M Soriano‐Gabarró are full‐time employees of Bayer AG (M Soriano‐Gabarró is the Head of Epidemiology at Bayer AG). V. Singh worked as an intern at Bayer AG in 2015 for a 6‐month period and is currently an employee of CSL Behring, Marburg, Germany. E. Artime is an employee of OXON Epidemiology, and N. Qizilbash is Head of OXON. V. Singh has no conflicts of interest. The project was conducted in collaboration with Oxon Epidemiology without any monetary assistance.

## Supporting information

Figure S1. Most frequently included European Union countries in cross‐sectional surveys evaluating the effectiveness of risk minimization measures.

Figure S2. Most frequent European Union countries for risk minimization effectiveness studies using secondary data sources.
