# Supplementary figures and images for: A review of studies evaluating the effectiveness of risk minimisation measures in Europe using the European Union electronic Register of Post‐Authorization Studies
Source: Pharmacoepidemiol Drug Saf. 2018 Apr 16;27(7):695–706. doi: 10.1002/pds.4434 (PMC6055865; doi:10.1002/pds.4434)

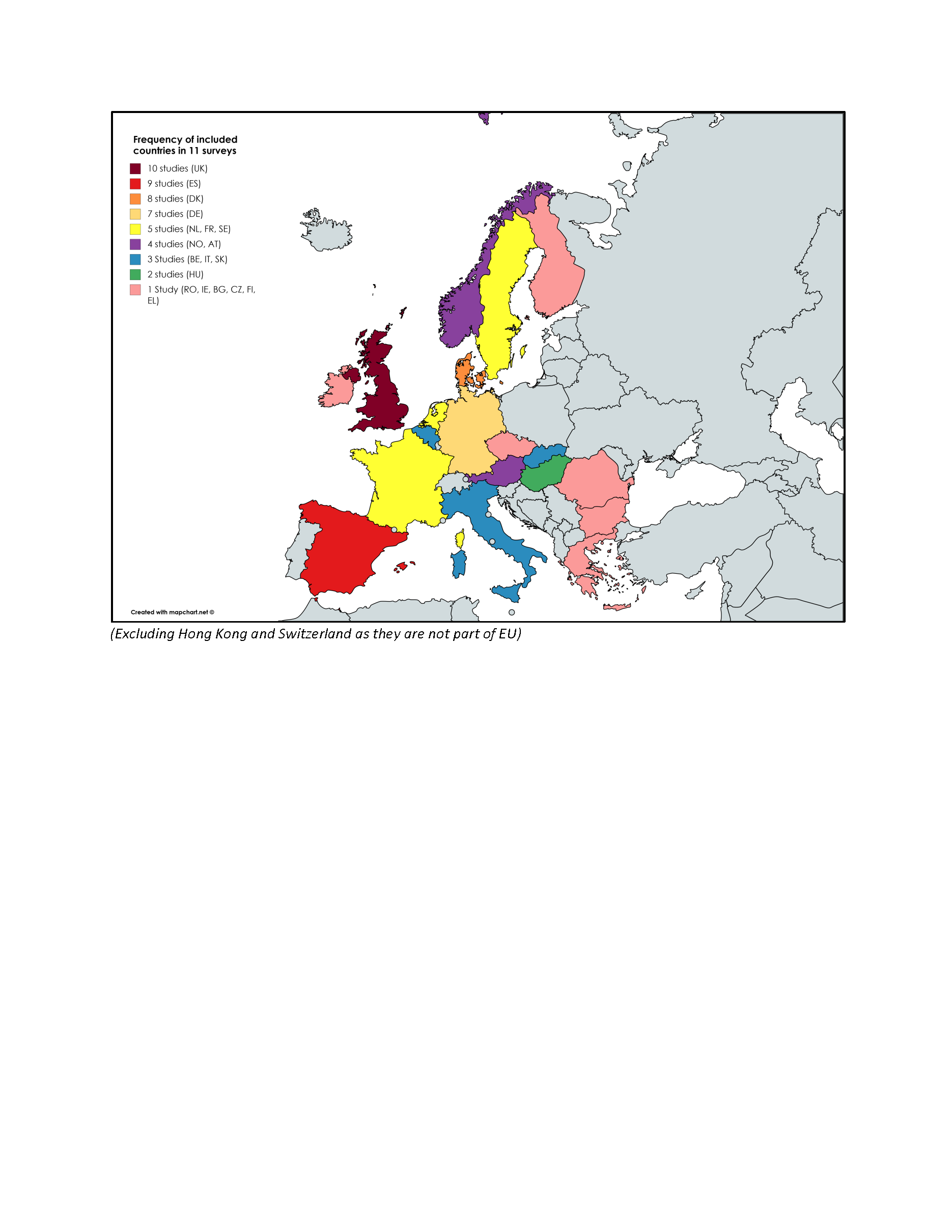

Supplement: Supplementary file 1 — Figure S1. Most frequently included European Union countries in cross‐sectional surveys evaluating the effectiveness of risk minimization measures. [file PDS-27-695-s001.tiff]

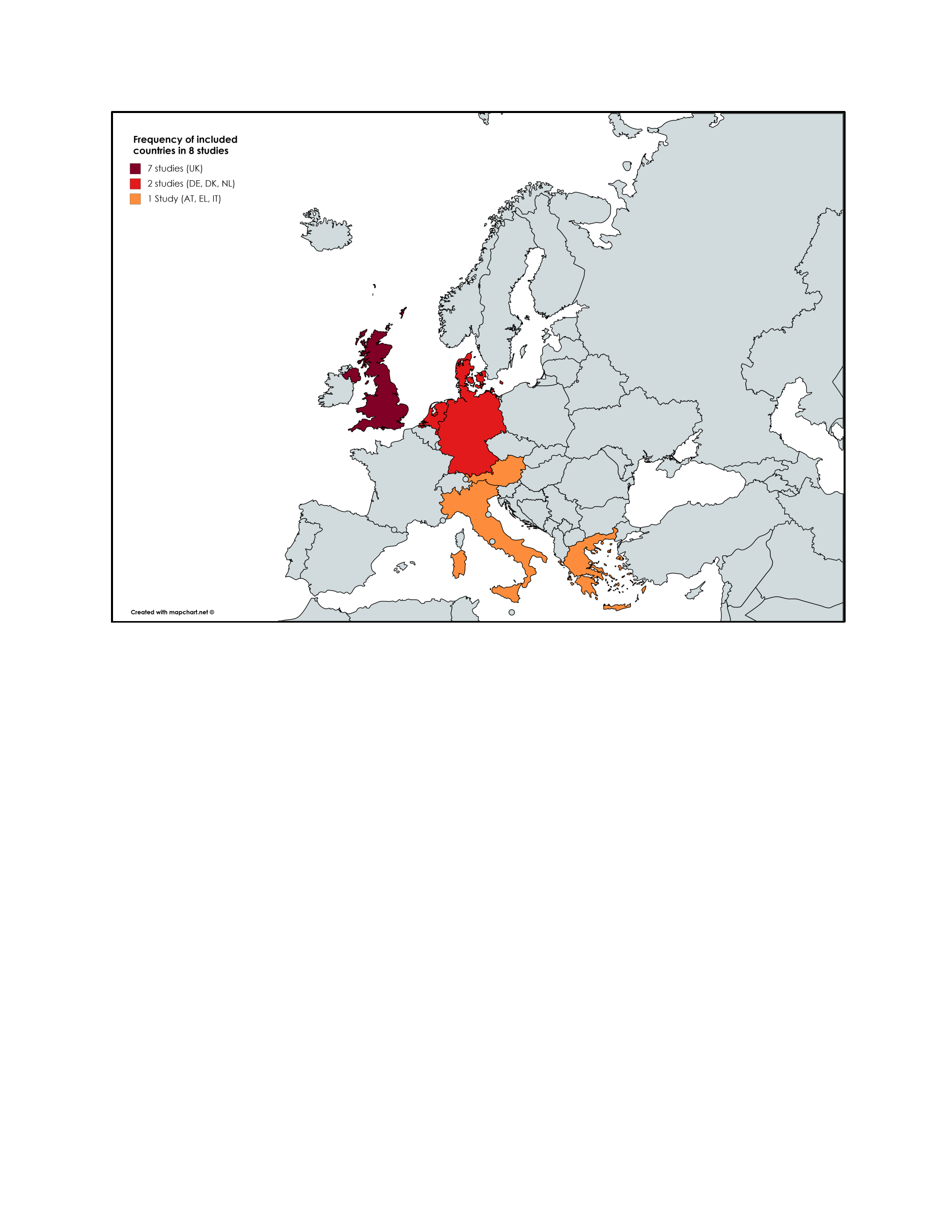

Supplement: Supplementary file 2 — Figure S2. Most frequent European Union countries for risk minimization effectiveness studies using secondary data sources. [file PDS-27-695-s002.tiff]
